# Audiological Service Delivery and Uptake in New Zealand Regional Areas

**DOI:** 10.3390/healthcare11233054

**Published:** 2023-11-28

**Authors:** Helen Boseley, David Welch, Ravi Reddy

**Affiliations:** 1 Section of Audiology, Faculty of Medical and Health Sciences, School of Population Health, University of Auckland, Auckland 1023, New Zealandd.welch@auckland.ac.nz (D.W.); 2 School of Health Sciences, Massey University, Auckland 0752, New Zealand

**Keywords:** audiology services, access barriers, regional, rural health, hearing loss, equity

## Abstract

Background: In New Zealand, as in many places, a significant proportion of the population lives outside the main urban centres. People living in regional areas have similar needs for audiological services as those living in urban centres; however, economic and geographical barriers can be a barrier to accessible services. The objective of this research was to explore factors that influence equitable audiological service provision and user uptake of services in regional areas of New Zealand. Methods: Fifteen participants who represented either audiological service users living in rural or non-urban areas (regional) or audiological service providers in these areas were recruited. Semi-structured interviews were conducted virtually and on average took forty minutes to complete. The interviews were transcribed and analysed using thematic analysis to identify themes and subthemes related to audiological service delivery and uptake. Results: Seven themes were identified. These are related to service provision, geographical barriers, and cultural appropriateness. Conclusions: This study provides a basis for understanding the challenges of delivering and accessing audiological services in non-urban areas in New Zealand, and in principle elsewhere. There is scope for future research to further understand policy directions needed to achieve equitable audiological service provision in regional areas.

## 1. Introduction

It is estimated that in 2016 approximately 20% of the New Zealand population experienced hearing loss, costing the country NZD 4.9 billion [1,2]. The likelihood of experiencing hearing loss increases among individuals aged over 65 years, and by 2061, this age group is anticipated to constitute a quarter of the total population, with rural areas expected to see the most significant rise [3]. Ageing and exposure to loud sounds typically cause permanent sensorineural hearing loss. The primary treatment involves amplification devices like hearing aids, enhancing audibility, speech perception, and utilizing residual hearing capabilities [4,5]. Since hearing levels tend to deteriorate over time, and the devices require maintenance, it is necessary for hearing aid users to have audiological care available. Given the economic and social costs associated with hearing loss, it is concerning to note that individuals often postpone seeking treatment for approximately a decade after its onset [6,7]. One barrier reported is that those needing assistance are not aware of services available and that audiological services should be more visible [8,9]. This lack of awareness is more common in rural areas where lack of audiological services and greater distances to available services lead to longer delays in rehabilitation and treatment [10,11,12,13]. As such, obstacles to access of audiological services in rural areas may remain throughout the patient journey, from seeking help to successful hearing health outcomes.

Approximately twenty percent of New Zealand’s population who reside in rural areas face difficulties in accessing healthcare and have poorer overall health outcomes compared to urban dwellers [14]. The 2023 New Zealand Rural Health Strategy report identified gaps in maternity care, urgent care, emergency services, and mental health services, which were exacerbated by workforce shortages and inadequate service provision in rural areas [14]. The report also highlighted service uptake challenges such as difficult referral processes, long wait times, inadequate access to acute mental health support and impractical appointment times that require extensive travel. Considering the difficulties that rural communities face in accessing a range of health services, it is plausible that similar challenges may exist in accessing audiological hearing health services.

In New Zealand, audiological services are provided through both the public sector (government funded) and through the private sector through a variety of mechanisms. However, audiological services, requiring specialized sound-treated rooms and bulky equipment, are predominantly located in large urban hospitals and private practices. Audiology departments provide fully funded audiological services and hearing technology for children and young people in full-time education, and new-born hearing screening services [15]. Fully funded services for adults are provided through public hospitals; however, most adults are not eligible for fully funded hearing aids and waiting lists can be up to two years. Therefore, services for adults are largely provided through private audiology clinics [1,16].

Access to healthcare services poses an ongoing challenge in regional areas of New Zealand, with populations facing specific barriers like insufficient transportation, limited telecommunications, high service costs, and restricted service provision. [17,18]. Evidence from Australia and the United States of America suggests inadequacies in audiological service provision and low uptake of services outside of major cities [11,13,19,20]. While factors such as financial costs, convenience, satisfaction, influence of social networks, and attitudinal beliefs have been reported to influence service delivery internationally [12,21,22], there has so far been limited research investigating accessibility barriers to audiological services in regional New Zealand. The aim of this study was to explore and identify factors related to the uptake of audiological services in rural communities and non-major urban areas in New Zealand. The findings of this research could inform future interventions aimed at increasing audiological service access and uptake and promoting hearing health in rural and regional communities.

## 2. Materials and Methods

### 2.1. Study Design

A phenomenological research design was used, with semi-structured interviews conducted to allow an in-depth exploration of the perceptions and personal experiences related to audiological service delivery in regional areas.

### 2.2. Participants and Recruitment

All participants were required to be over 18 years old and proficient in English. In New Zealand, geographic areas are defined by the Statistical standard for geographic areas 2023 [23]. The territorial boundaries are defined as major urban area (100,000+ residents), large urban area (30,000–99,999 residents), medium urban area (10,000–29,999 residents), small urban area (1000–9999 residents), and rural areas (200–999 residents with at least 200 residents per square kilometre) [23]. Participants had to live in a rural and non-major urban area, collectively referred to as “regional” areas henceforth. Private and public audiological service providers were required to offer services to regional communities in New Zealand. Purposive sampling was used to recruit participants through a private audiology service provider mailing list, and service providers were recruited via advertisements by the New Zealand Audiological Society. Those who volunteered were contacted by the primary author to provide a participant information sheet and were given an invitation to participate. Informed written consent to take part in the study was obtained from all participants prior to their participation. All participants interviewed were offered a NZD 20 grocery voucher at the end of their interview as a show of appreciation and respect for their time. A total of 15 participants were recruited, consisting of 7 service users, 4 private sector audiologists, and 4 public sector audiologists. Participants were recruited until thematic saturation was achieved and no new information was emerging [24]. Ethics approval for the study was received from the Auckland Health Research Ethics Committee (reference number AH3057).

### 2.3. Data Collection

Semi-structured interviews were conducted by the primary author over the phone or virtually via video calls. An interview guide was developed to allow a wide coverage of topics related to the accessibility and uptake of audiological services. The guide was developed by the primary author and based on feedback from the co-authors. Revisions were made before confirmation of the final version (Table 1 and Table 2). Interviews were between 35 to 65 min long, recorded on Zoom and phone and transcribed using intelligent verbatim by the primary author. No one else was present during the interviews, to maintain privacy. Participants were offered the opportunity to involve family or friends as support, but none did. All transcripts were offered to the participants for comment or correction; however, no alterations or repeat interviews were needed. Data analysis was conducted by the primary author with reviews conducted by co-authors. The primary author is a female New Zealand European who grew up in a small forestry town, has also lived in major urban areas, and understands that there are specific challenges to accessing healthcare from smaller and rural communities in New Zealand.

### 2.4. Data Analysis

Interview transcriptions were de-identified and imported to the qualitative data analysis software programme QSR International NVivo (Release 1.3). Thematic analysis was undertaken using the six-phase steps described by Braun and Clarke [25]. This included familiarisation with data content and the generation of codes that described features of the data. The primary author did the coding of the transcripts, and the co-authors reviewed the coding framework to corroborate the codes and reviewed and edited themes and subthemes for suitability and labelling. The coding framework related to factors influencing the uptake of audiological services in regional areas. We explored if other terms could offer a better description of the themes, which were derived from the data. These themes and codes were validated through the consensus of the research group. None of the participants requested a summary of the findings, even though it was offered during the interviews.

## 3. Results

Seven themes were identified from service user and service provider interview transcripts which related to the accessibility of audiological services in regional areas. These were (1) lack of services, (2) distance to services, (3) transportation, (4) staff shortages, (5) wait times, (6) access to primary healthcare, and (7) cultural barriers. Proper names such as those of specific towns or companies have been removed from quotes to preserve participant confidentiality.

### 3.1. Service Users

#### 3.1.1. Lack of Services

Participants felt there was an absence of services in regional areas. These not only related to healthcare services but general community services readily available in urban areas. There is a perception that those who do not live near the city are considered rural or regional even if some are only a short distance away. In addition, the participants felt that services are set up in proportion to the population of areas.

“*People think we live in the sticks… we’re nine km from what we call [name of rural settlement], and the only thing that’s down there now, there’s a golf club and a school*”.

“*I did do a search and found they [a private audiology provider] weren’t in [name of small urban area] anyway*”.

“*In the bigger cities, you can go and book in for your hearing or if you need your ears syringed, you can get that done pretty much straight away. Down here probably because there’s not enough people, they get in a person from [name of large urban area] every month to come down*”.

#### 3.1.2. Distance to Services

Audiological services are predominantly located in major urban areas, and participants confirmed the distance from regional areas was an accessibility barrier. The time to get to service providers can be perceived as a barrier to accessing services.

“*For audiology, I would say it’s quite localized to the bigger towns… it’s a long drive… with somebody living in [name of a rural area] … they can’t just drive down the road to the audiology place*”.

“*…we’re not the furthest away from [name of large urban area] by any means. We are an hour and 15, or an hour 20 min away… but we live on a one-way road and there’s about another 40 km to go. There are people who have a further 40 km to travel. And it’s quite a rough road… So that’s just one issue*”.

##### Acceptance of Remoteness

On the other hand, participants also expressed that people living in regional areas expect to travel long distances to audiological services. They spoke of adaptability and willingness to overcome distance barriers.

“*I mean it [travelling long distances to audiological services] would be just like going to the doctors, or going to the physio, the dentist. You know, that’s life for them, you know*”.

However, participants were reluctant to travel large distances for short appointments but did not mind the distance if they felt the service was worthwhile.

“*You kind of wonder whether you’re going all that way and all that way back just for a five-minute appointment… thorough examination is certainly well worth it*”.

#### 3.1.3. Staff Shortages

Service users identified staff shortages and staffing challenges for audiology in regional areas as an accessibility barrier. It has been reported that there are problems related to service sustainability in the event of local audiologists leaving.

“*It’s [audiology services] pretty much reduced… I think they have had an audiologist come through on a spasmodic basis... There used to be a guy here, who was full-time… And then he retired, and they did have some limited hearing tests and syringing for blocked ears, and they would have an audiologist come through probably once a month*”.

In addition, service users believed more staff were needed to provide adequate and timely service for the population.

“*…[if] they haven’t been able to get enough audiologists to fill all the appointments, then that’s a way the service could be improved—by increasing the number of trained people*”.

### 3.2. Service Providers

#### 3.2.1. Lack of Services

Audiological service providers felt their outreach clinics to rural areas lacked resources or commitment to provide adequate services. These suggest priorities in terms of business models and population density may influence resources and service provision.

Private service provider:

“…*they [the private service provider] used to have a satellite clinic at [small urban area] … We no longer do that*…”.

Public service provider:

“*We are quite limited in terms of other services we can provide [in rural hospitals] … The audiologist has the choice to see a patient close to home in one of the rural hospitals or in the bigger centre in [a major urban hospital], where they’ve got better equipment and better rooms*”.

#### 3.2.2. Travel to Service Providers

There was agreement between service providers that distance to audiology clinics was a barrier to accessing hearing care in regional areas. The time to get to service providers could possibly demotivate people to seek care for their hearing. However, there was a perception that regional communities, due to their location, accept that travelling long distances to access services is normal. In addition, transportation limitations added to the burden of accessing services.

##### Distance to Services

Public service provider:

“*…people in [regional area] kind of expect it [long distances to services] to a certain extent, because that’s all they’ve ever known*”.

Private service provider:

“*They feel they probably don’t have access, but they’re so used to it that they’re prepared to travel*”.

##### Social Inequities

Public and private service provider groups described a lack of public transport from regional areas and patients needed to either use their own private transport or were reliant on family to drive them to appointments. These include the cost of fuel, the needs of old people, and the inadequacy of the systems designed to help people mobilise.

Private service provider:

“*In rural areas… public transport is not the best. So, they would have to rely on someone else. Their daughter, their son, their grandkids*”.

Public service provider:

“*In [small urban area] where there’s no public transport system, you’re reliant on patients driving themselves to services*”.

Both service provider groups also commented on the additional barriers of using private transport such as access to private transport, cost of petrol, parking, and difficulty driving.

Private service provider:

“*…most elderly people don’t like travelling at certain times of the day, or they don’t like travelling longer distances… they’ll get stuck in traffic or they get tired*”.

Public service provider:

“*We’ve got people who live 17 km away who couldn’t come in because they haven’t got transport… and if you’ve ever tried to find a car park at [major urban hospital], it’s a nightmare*”.

Public service provider:

“*If you’ve got poverty issues in some areas… families simply don’t have the petrol to come*”.

Public service providers discussed the National Travel Assistance Scheme which supported families to pay for transportation to hospital appointments. The funding was retrospective and, therefore, it did not overcome the affordability barriers of private transport.

Public service provider:

“*…the way that it works with travellers [national travel assistance], you get re-compensated for it. So, you have to front up with the money to start with*”.

#### 3.2.3. Staff Shortages

Service providers identified staff shortages and staffing challenges for audiology in regional areas as an accessibility barrier. There is agreement that regional areas are under-manned to provide audiological services.

Private service provider:

“*That’s always been an issue with audiology. Getting fully qualified audiologists in these more regional towns*”.

Public service provider:

“*I don’t know if you know much about the [name of region] situation but there isn’t an audiologist there. There’s never been an audiologist*”.

The main explanation service providers had for the staffing shortages in regional areas was the unwillingness of audiologists to live in regional areas. There is a perception that audiologists are not attracted to regional areas and prefer to operate in high-density urban areas.

Private service provider:

“*Most of the clinicians are in Auckland, Wellington, and Christchurch, which are the main big hubs… there’s a lot of audiologists who want to remain in Auckland… not a lot of clinicians are willing to move to rural areas*”.

Public service provider:

“*Kiwis [New Zealanders] don’t want to work in [a regional area] … It’s really hard to attract them [audiologists] to the regions*”.

This was believed to be because the training institutions for audiology are located only in Auckland and Christchurch. As such, New Zealand audiologists are generally from these cities and prefer to remain close to home. Additionally, regional areas were not seen as exciting places for young people to live, and there was a lack of employment opportunities for audiologists’ partners.

Private service provider:

“*…audiology [Masters programmes] are only in Canterbury and in Auckland. It mostly attracts people who are closer to those areas… I don’t think a lot of people want to leave their friends and families behind*”.

Public service provider:

“*Kiwi [New Zealanders] graduates want to work in Auckland, and they want to work in Christchurch… because that’s where most of them are from… they want to go somewhere where there’s more opportunities and things for young people to do, as well as considering what their partners do for work*”.

Public service providers expressed their desire to see larger centres with more staff take responsibility for smaller areas when they experience staff shortages and felt the government’s new health delivery plan was a step towards this.

Public service provider:

“*What I would like to see is more reliance on the bigger centres who have consistent staff for longer… have their expertise spread over into these rural centres. I think we may be seeing a move towards that*”.

#### 3.2.4. Waiting Times

Longer waiting times for audiological services were experienced by those living outside of major urban areas. Private service providers thought that the long wait times may influence people to choose not to seek help, while public service providers thought the longer waiting times may be a reason for patients and families to forget their appointments, thereby delaying access to services.

Private service provider:

“*In Auckland you would probably have to wait maybe a few days… compared to waiting three or four weeks [in regional areas]*”.

Private service provider:

“*Once they [patients] have made that decision to do something, they just want to get on with it. If they can’t get seen, then they’ll think, ‘oh well. Forget it*”.

Public service provider:

“*…there’s a long waitlist, some families may have forgotten about the appointment. It gets to the back of their radar… it’s forgotten about, or it’s not attended*”.

However, private service providers also believed patients who lived in regional areas expected to wait longer for appointments just as they perceived the acceptance of travelling long distances to access services.

Private service provider:

“*If you retire to [a small urban area] or [another small urban area], or somewhere in between, then I think you’d expect more of a wait because that’s quite rural*”.

#### 3.2.5. Access to Primary Healthcare

The lack of access to General Practice (GP) services in rural areas is a barrier to public audiological services. This is because public audiological services are often via GP referrals. This was only discussed by public service providers.

Public service provider:

“*Whether rurality would affect someone’s ability to go to the GP… I don’t know how well GP practices are spaced out and people can access them to get audiology*”.

#### 3.2.6. Cultural Barriers

Both service provider participant groups identified that audiological services were less accessible for Māori. This was attributed to a Western model of care and experiences of being marginalised by government services, which has led to mistrust in public audiological services.

Private service provider:

“*…they [Māori] didn’t trust you. And I think that’s generational… Going back generations those services haven’t been adequately provided to those people, and now suddenly you’re saying, ‘Hey, come to the clinic, do what I say*”.

Public service provider:

“*…we have that westernized model that people don’t fit into…You’ve got a lack of cultural competence… that will influence whether a family engages with the service… I think that probably speaks to why we have a non-attendance rate for New Zealand European that is below 10%, whereas for Māori, you are looking at 20% to 30%*”.

## 4. Discussion

The key barriers to accessing audiological services in regional communities identified in this research were (1) lack of services, (2) distance to services, (3) social inequities, (4) staff shortages, (5) wait times, (6) lack of access to primary healthcare, and (7) cultural barriers. On the other hand, there was a theme about adaptability and willingness to cope with barriers if they are not insurmountable.

Participants said that most hearing healthcare services were available only in major urban areas and participants noted barriers to accessing those services due to social inequities. This supports existing evidence that health service capacity and delivery in New Zealand have been greater in urban areas [26,27]. In addition, findings from the United States have shown higher proportions of audiologists in urban areas [13]. The limited service provision in regional areas is exacerbated by poor public transportation systems and decreased private transport ownership due to greater socioeconomic deprivation in these areas [28]. Participants expressed willingness to travel greater distances for services they deemed “worthwhile”. Therefore, promoting audiological services that underscore their comprehensiveness could enhance uptake in rural communities. Additionally, offering shorter and less technical services through internet connections may be beneficial, while longer appointments requiring technical resources could be managed cautiously.

A shortage of staff contributed to the absence of audiological services and prolonged waiting times in regional areas. This aligns with the recruitment and retention challenges observed in rural and provincial areas across various health disciplines in New Zealand, including general practice, nursing, pharmacy, and optometry [29,30]. A comprehensive, long-term strategy is essential to address audiological service provision in regional areas, mirroring the plans established for the growth of GP practices. In response to rural GP shortages, The Royal New Zealand College of General Practitioners developed a 10-year strategic plan to strengthen and sustain rural GP practice [31]. Financial incentives for audiologists to work in rural and regional areas are likely to attract staff looking for an attractive long-term option. Furthermore, rural and regional areas are not seen as desirable places to live due to a lack of economic development outside of major urban cities [32]. Regional development is therefore crucial to attract and retain audiologists to regions outside of the main cities where there are staffing shortages. In addition, developing the rural and regional GP workforce could indirectly improve access to public audiological services. This is because public audiological services require a GP referral, and currently, 74% of New Zealand’s GPs work in an urban-based clinic [27]. This was highlighted by the current research finding that the lack of GP coverage and access to primary health care in rural areas is a barrier to accessing public audiological services. Increasing the number of audiologists in regional areas may be achieved by either training a larger workforce or prioritizing the immigration of audiologists to the country, driven by economic pressures. The only two New Zealand training institutions for audiology are in the major urban areas of Auckland and Christchurch. These institutions may likely attract students who are local to these areas and would often want to remain there when they graduate. This is in line with evidence that social and family supports are important factors when individuals make employment decisions [33,34]. This is supported by evidence that students from regional areas are more likely to work outside of major urban cities when they graduate [35]. This suggests that strategic decisions to attract students from regional areas must be an important policy priority for the country. An example of a strategic policy direction is a preferential admission quota to attract regional students into the medical degree programmes in New Zealand universities.

The recent reform of the public health system in New Zealand recognises the challenges of accessibility to healthcare in rural areas and has seen a shift to a locality network model [36]. This is to ensure healthcare is designed to meet the unique needs of these communities. Public service providers in this study were interviewed before the announcement of the reform. Nevertheless, they expressed a preference for a health system reform which could ensure consistency of healthcare access in regional areas where there are few staff and turnover is high. This indicates the reform will be well received among public service audiologists as the new focus aims to better support health professionals in rural areas.

There was a perception that audiological services may not be culturally appropriate for individual communities such as indigenous Māori, due to the Westernised model of healthcare. The biomedical model is frequently used in Western models of healthcare and focuses on biological causes of illness while giving less attention to psychological, social, and spiritual influences [37,38]. This has implications for service provision, especially given that social inequities are more likely to influence Māori people’s access negatively.

The service-user group in this research had experience using audiological services. The recruitment strategy to use service-provider client mailing lists introduced sampling bias. Therefore, perceptions were unable to be explored among those with hearing loss who had not accessed audiological services. It would be valuable to understand what prevents such people from accessing services, as their views may differ from those in our sample. Previous research implies that rural New Zealanders tend to prefer approaches that they are used to, so views from this participant sample may not be reflective of all rural New Zealanders who may prefer traditional in-person services [39]. The research did not explore demographic differences such as age, gender, ethnicity, or economic status in relation to audiological service provision in rural areas. In addition, there were no paediatric patients or caregivers of children with hearing loss included in the patient sample and, therefore, views from paediatric populations remain unexplored. A larger sample size comprising stratified groups would help explain the variability and allow for the generalizability of findings in future research.

Lastly, interviews were conducted via either the telephone or an internet video link rather than in-person, which likely introduced a sampling bias towards people with internet connections, who may tend to be wealthier and to have a more modernist view than people without. It is reasonable to assume that participants in our sample were already comfortable with technology and had access to a device or home phone. Patients without a device, who are uncomfortable with technology, or who struggle with using a landline phone due to their hearing loss were unlikely to participate. They were therefore unintentionally excluded from the sample. These people may have different perspectives from those who participated, which limited the range of views captured in this study. The sampling bias in this research could have been mitigated to some extent by conducting focus group discussions with separate service user and service provider groups as part of a triangulation approach. However, the findings of this research can inform future research targeting audiological services in rural and regional communities.

## 5. Conclusions

This study identified accessibility barriers to audiological services in regional areas in New Zealand. The findings provide a basis for understanding the challenges of accessing audiological services from these areas and suggest that audiological services are not being adequately provided in these areas. The 2023 New Zealand Rural Health Strategy report highlighted limited allied health services in rural areas such as dental care, pharmacy services, physiotherapy, and occupational therapy [14]. While the report conspicuously overlooks the provision of audiological services in rural areas, our findings offer compelling evidence that audiological services encounter comparable challenges to those identified in other health service areas. To improve audiological service provision, interventions may involve increasing the number of trained audiologists, expanding services in regional areas, and improving public transportation to areas with greater service availability. Efforts should also focus on establishing culturally acceptable services, enhancing general practitioner (GP) coverage in rural regions, promoting regional economic development, and implementing strategies to attract regional students to audiology training programs. These measures collectively aim to develop and strengthen the regional audiological workforce while improving overall accessibility to audiological services in these areas. Future directions for research could build on the current findings by quantifying these perceptions and collecting demographic information to explore patterns between age groups, ethnicity, gender, and regional variations. In addition, new models of service delivery such as tele-audiology and its feasibility in regional communities could be investigated. There is potential for the development of strategies and interventions aimed at achieving equity access to regional audiological services.

## Figures and Tables

**Table 1:** Interview guide for service users.

What hearing health services are you aware of?
How long was it from when you first started noticing you had a hearing loss before you sought help for your hearing? If there was a delay, what delayed you from seeking help for your hearing?
Have you addressed your hearing loss? If yes, what motivated you to addressing your hearing loss? If no, what has prevented you from addressing your hearing loss?
What do you think motivates or prevents other people in your community from taking up hearing-health services?
In what ways do you feel hearing-health services in your community could be improved?

**Table 2:** Interview guide for service providers.

How do you perceive the accessibility of hearing-health services in small urban/rural areas?
How do you think these communities perceive the accessibility of hearing-health services?
Why do you think people in small urban/rural communities do/don’t seek help for their hearing? Do you think people in these communities are less likely to ask for help about their hearing?
What do you think motivates or prevents other people in these community from taking up hearing-health services? Do people delay seeking support? Why?
In what ways do you feel hearing-health services in small urban/rural areas could be improved?

## Data Availability

The data presented in this study are available in article quotations reported in the results.
